# Analysis of influencing factors on Turner syndrome combined with autoimmune thyroid disease

**DOI:** 10.3389/fendo.2025.1547430

**Published:** 2025-08-06

**Authors:** Yingmei Cen, Wei Wei, Yinchun Huang, Yiying Qi, Gendie E. Lash, Li Li

**Affiliations:** ^1^ Department of Obstetrics and Gynecology, Guangzhou Women and Children’s Medical Center, Guangzhou Medical University, Guangzhou, China; ^2^ Guangzhou Institute of Pediatrics, Guangzhou Women and Children’s Medical Center, Guangzhou Medical University, Guangdong Provincial Clinical Research Center for Child Health, Guangzhou, China

**Keywords:** Turner syndrome, autoimmune thyroid disease, thyroid autoimmunity, chromosomal karyotype, pediatric care

## Abstract

**Background:**

Autoimmune thyroid disease (AITD) is the most common autoimmune disease in patients with Turner Syndrome (TS). There is a high prevalence of AITD in TS patients, it has an early age of onset and can present as severe thyroid dysfunction. The specific etiology of AITD in TS is not clear and may be associated with sex chromosome-related genetic defects, immune dysfunction, or sex hormone imbalance due to ovarian insufficiency. The aim of this study was to investigate the prevalence and related influencing factors of AITD in Chinese patients with TS.

**Methods:**

In total 63 female patients aged 14–32 diagnosed with TS received titer of thyroid autoantibodies and thyroid function examinations, including thyroid stimulating hormone (TSH), free tetraiodothyronine (FT4), free triiodothyronine (FT3), total thyroxine (TT4), total triiodothyronine (TT3), thyroid peroxidase antibody (TPOAb) and thyroglobulin antibody (TgAb). The effects of age, body mass index (BMI), karyotype, fasting insulin, blood lipids and other factors that may affect thyroid function were analyzed, and the possible risk factors associated with AITD in TS patients explored.

**Results:**

Of the 63 TS patients, 24 (38.10%) had normal thyroid function and 39 (61.90%) patients had TgAb and/or TPOAb. Among the 39 women with positive thyroid autoantibodies, 10 had abnormal thyroid function, including 3 with hyperthyroidism and 7 with hypothyroidism. Patients with isochromosome X had an increased risk of developing AITD.

**Conclusions:**

The risk of AITD with TS correlates with the chromosomal karyotype, patients with isochromosome X have an increased risk of AITD. Patients with positive thyroid autoantibodies have a higher risk of thyroid dysfunction.

## 1 Introduction

Turner Syndrome (TS), also known as congenital ovarian hypoplasia syndrome, is a sex chromosome disorder caused by abnormalities in the number or structure of the X chromosome, with a prevalence rate of approximately 1/2500-1/4000 in live-born female infants (1, 2). The most common karyotype of TS is 45, X, accounting for about 30%-40% of all karyotypes, followed by mosaic patterns (45, X/46, XX), accounting for about 20%-30%, and the rest are X chromosome abnormalities (insertions and deletions). In addition, Y chromosome material is present in about 5% of TS patients and chromosomal markers (segments originating from the X or Y chromosome) are present in 3% of TS patients (3, 4). TS typically presents with short stature, ovarian insufficiency and congenital malformations, and is often accompanied by a variety of autoimmune disorders, such as autoimmune thyroid disease (AITD), diabetes mellitus, juvenile idiopathic arthritis, inflammatory bowel disease, celiac disease, and psoriasis. Of these, AITD is the most common autoimmune disease in patients with TS (5).

The relationship between TS and thyroid disease was first suggested by Atria et al. (6), who found a large lymphocytic infiltration in the thyroid gland in the autopsy report of a patient with TS. A meta-analysis of cross-sectional studies in Europe showed that the prevalence of AITD in TS patients ranged from 10.5% to 67.5% (7). The prevalence of different studies varied greatly, which may be due to differences in age, karyotype, sample size and race of the subjects. The etiology of AITD in TS has not been widely studied, it may be associated with genetic factors related to sex chromosomes and sex hormone levels (8–10). Previous studies have shown that the deletion of related genes caused by haploinsufficiency of the X chromosome plays a key role in TS combined with thyroid disease, and that at least 10 genes on the X chromosome were involved in immunomodulatory effects, these genes provide important clues to our deeper understanding of the mechanisms of TS combined with thyroid disease (11–13). AITD also occurs more frequently in TS females with isochromosome X (14, 15). Genes located on the long arm of the X chromosome may play a significant pathogenic role in the development of autoimmune diseases, while an imbalance between loci on the short and long arms of the X chromosome may elevate the risk of immune dysfunction (16). Bakalov et al. (8) reported that lymphocytic thyroiditis is greatly increased in both women with TS and primary ovarian insufficiency (POI), suggesting that factors associated with ovarian insufficiency per se promote this form of autoimmunity, and the absence of a normal second X-chromosome further contributes to increased autoimmunity in TS. TS patients often have concomitant metabolic disorders. Studies have shown that there is a potential link between multiple metabolic abnormalities and AITD. Obesity can lead to changes in thyroid hormone activity, malnutrition or nutritional deficiencies can be the cause of thyroid disorders, and AITD is associated with abnormalities in glucose metabolism, resulting in an increased risk of type 1 and type 2 diabetes mellitus (17). In a longitudinal cohort study in Korean women dyslipidemia was shown to be a risk factor for hypothyroidism (18). However, there is currently a lack of national and international studies investigating the potential association between metabolic abnormalities and AITD in TS women.

Thyroid dysfunction in children can have an impact on both physical and mental development. Hypothyroidism or subclinical hypothyroidism may increase the risk of cardiovascular disease, including coronary atherosclerosis and acute coronary syndromes (19, 20). Therefore, a heightened awareness of AITD may lead to early diagnosis and treatment, thereby preventing adverse effects on growth, intellectual development, and even the cardiovascular system. Here, this study aimed to investigate the factors that affect AITD in TS patients, including age, chromosomal karyotype, body mass index, lipid levels, calcium levels, phosphorus levels, and fasting insulin levels.

## 2 Materials and methods

### 2.1 Clinical characteristics

Sixty-three female patients diagnosed with TS in the Department of Gynecology and Endocrinology, Guangzhou Women and Children’s Medical Center affiliated to Guangzhou Medical University were recruited and confirmed by chromosome karyotype analysis of their peripheral blood (21). Patients who had not received growth hormone therapy in the last three years and were taking hormone replacement therapy were included. Patients who refused to cooperate or were unable to undergo thyroid examination were excluded. All patients denied a history of radiation exposure, hypothalamic or pituitary disease, a family history of thyroid disease, smoking and drinking history. General information and clinical data of the patients were collected, including age, height, weight, body mass index (BMI), chromosome karyotype, thyroid function, thyroid autoantibodies, blood lipid concentrations, and hormone treatment status. This study was approved by the Ethics Committee of Guangzhou Women and Children’s Medical Center, and all participants or their guardians signed an informed consent form.

### 2.2 Laboratory examinations

Fasting venous blood samples were taken from all subjects. Peripheral blood was collected into inert separating gelatinizing tubes, serum collected and stored at −20°C until required for analysis. Thyroid stimulating hormone (TSH), free tetraiodothyronine (FT4), free triiodothyronine (FT3), total thyroxine (TT4), total triiodothyronine (TT3), thyroid peroxidase antibody (TPOAb), thyroglobulin antibody (TgAb), total cholesterol (TC), triglyceride (TG), high-density lipoprotein cholesterol (HDL-C), low-density lipoprotein cholesterol (LDL-C), calcium (Ca), phosphorus (P), and fasting insulin (FIns) were measured in the serum of all subjects. Thyroid function and fasting insulin were measured by the electron chemiluminescence immunoassay in the Abbott I2000 analyzer, and calcium, phosphorus and blood lipid levels of all subjects were measured by the Hitachi 7600–200 analyzer.

### 2.3 Diagnostic criteria

#### 2.3.1 TS diagnostic criteria

Karyotype analysis of peripheral blood is the gold standard for the diagnosis of TS and usually requires karyotype analysis of 30 standard cells (21). If <10% of cells were karyotyped as chimeric and chimerism was strongly suspected, at least 50 interphase and more mid division cells were counted or fluorescence *in situ* hybridization analyzed to exclude chimerism. For the majority of cases, karyotype type is sufficient to confirm the diagnosis of TS. If TS is strongly suspected clinically, but the result of the initial karyotype type is normal, a second karyotype type should be performed using different tissues, such as skin (fibroblasts), bladder epithelial cells in urine samples or buccal mucosal cells (22).

#### 2.3.2 Diagnostic criteria for AITD

1AITD is an inflammatory condition of the thyroid gland caused by an autoimmune response, which triggers lymphocytic infiltration. The condition is characterized by the presence of thyroid-specific antigenic cells and thyroid autoantibodies in the circulation. The diagnosis was based on the guidelines determined by the Chinese Medical Association.

### 2.4 Statistical analysis

The SPSS 26.0 software package was used for statistical analysis of all data. The measurement data with normal distribution is represented by mean ± standard deviation, and the comparison between the two groups was performed by t test. Counting data are expressed as the number of cases and percentages and compared using chi-square test or Fisher’s exact test. *P*<0.05 was considered as statistically significant.

## 3 Results

### 3.1 Basic characteristics

A total of 63 patients aged 14–34 years with a diagnosis of TS were included in the study, and the basic characteristics of the study participants is shown in **Table 1**. Karyotype analysis of peripheral blood was performed in all patients, in total 34 (53.97%) patients had a chromosome karyotype of 45, X, 12 (19.05%) patients had isochromosome X, the rest had other chromosome karyotypes, as detailed in **Table 1**.

**Table 1:** Basic characteristics of the study participants.

Subject	Value
Age (years)	20.92 ± 4.30
Height (cm)	146.37 ± 7.67
Weight (kg)	47.77 ± 10.54
BMI (kg/m^2^)	22.16 ± 4.10
Karyotype	N (%)
45, X	34 (53.97%)
45, X/46, X, i(X) (q10)	9 (14.29%)
46, X, i(X) (q10)	2 (3.17%)
46, X, i(X) (q10), inv (9) (p12q13)	1 (1.59%)
45, X/46, XX	4 (6.35%)
45, X/46, X, r(X)	2 (3.17%)
45, X/46, X,+mar	2 (3.17%)
45, X/46, XY	2 (3.17%)
45, X/47, XXX	1 (1.59%)
46, X, del (X)(p11)	1 (1.59%)
46, X, idic (X), (p22.1)	1 (1.59%)
46, X, t(X;14) (q21.2;q32.1)	1 (1.59%)
45, X/46, X, del (X), (q22q28)	1 (1.59%)
45, X/46, X, idic (X) (q10)	1 (1.59%)
45, X/46, X, +mar/46, X, r(X) (p22q26)	1 (1.59%)

BMI, body mass index; inv, inversion; idic, isodicentric chromosome; del, deletion; mar, marker chromosome.

### 3.2 Thyroid function examinations

**Figure 1:**
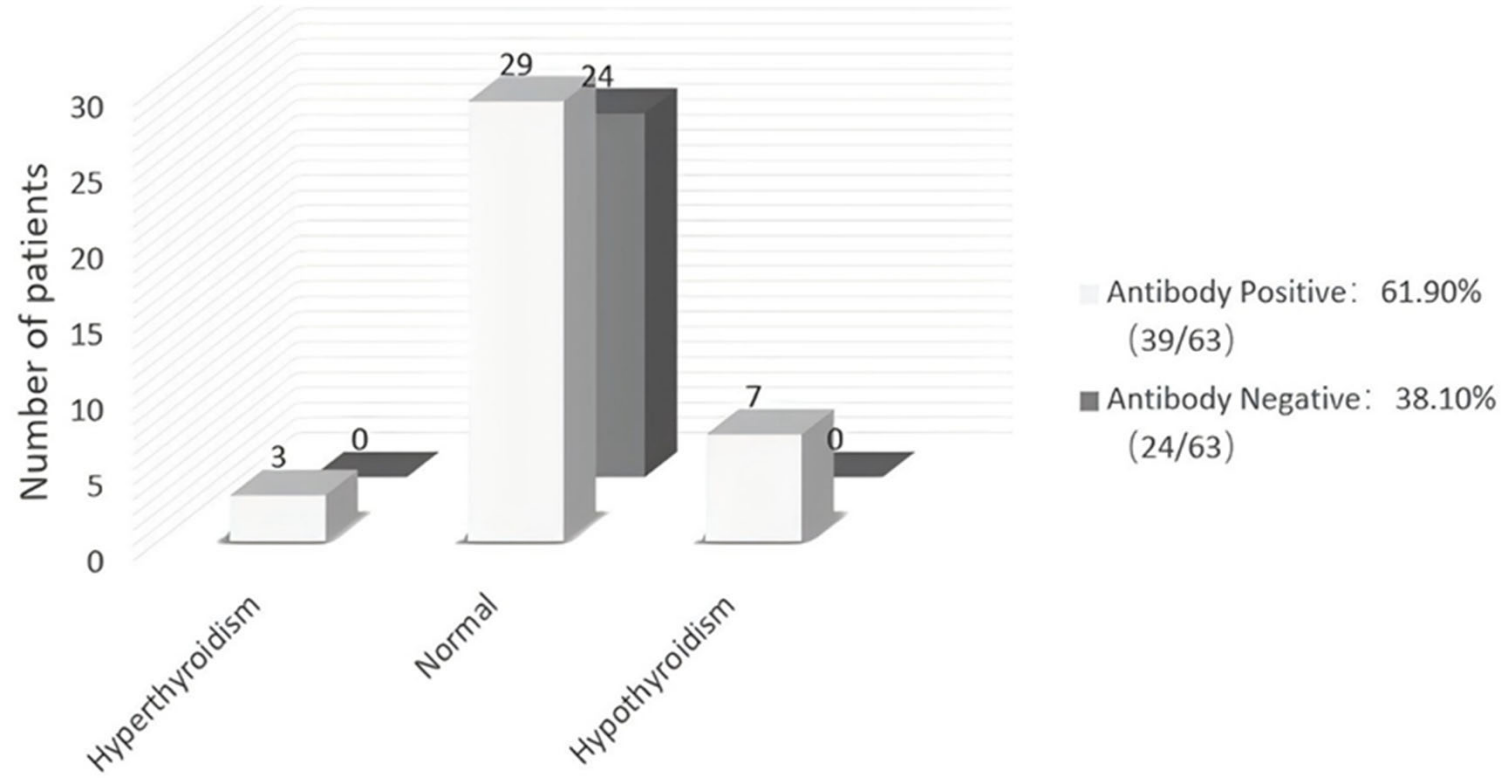
Thyroid function and thyroid autoantibodies in 63 patients with Turner Syndrome. Rates of hyper-, hypo-, and normal thyroid function in TS patients with or without autoantibodies. The numerals in the columns are the number of patients within each group.

Thyroid function was examined in all 63 patients with TS. Thyroid autoantibodies were undetectable in 24 of 63 TS patients (38.10%), and thyroid function was normal in all these women. Of the 39 TS patients with thyroid autoantibodies (61.90%), 3 had hyperthyroidism, 7 had hypothyroidism and 29 had normal thyroid function (**Figure 1**).

### 3.3 Risk factors for AITD in TS subjects

The results showed that AITD can occur in any type of karyotype, but in the group of isochromosome X, the percentage of AITD in patients with TS was 91.67%, which was significantly higher than the groups of 45, X and other alterations, which were 61.76% and 41.18%, respectively. There was a statistically significant difference in the rate of AITD among the three karyotype subgroups (*P*<0.05). Patients with the isochromosome X karyotype had a higher risk of AITD (**Table 2**).

**Table 2:** The prevalence of AITD according to karyotype.

	Number of negative thyroid autoantibodies [n (%)]	Number of positive thyroid autoantibodies [n (%)]	χ2	*P*
Type of karyotype			7.605	0.02
45, X	13 (38.23%)	21 (61.76%)		
Isochromosome X	1 (8.33%)	11 (91.67%)		
Other alterations	10 (58.82%)	7 (41.18%)		

Prevalence of AITD is calculated over the total number of patients per karyotype group.

Of the patients in the group positive for thyroid autoantibodies, 29 had normal thyroid function and 10 developed thyroid dysfunction. There was no statistically significant difference in the rate of thyroid dysfunction between the groups of euthyroidism and dysthyreosis based on chromosomal karyotype (**Table 3**).

**Table 3:** The prevalence of dysthyreosis according to karyotype.

	Euthyroidism number [n (%)]	Dysthyreosis number [n (%)]	χ2	*P*
Type of karyotype			1.738	0.4
45, X	17 (80.95%)	4 (19.05%)		
Isochromosome X	8 (72.73%)	3 (27.27%)		
Other alterations	4 (57.14%)	3 (42.86%)		

Prevalence of euthyroidism is calculated over the total number of patients per karyotype group.

In addition, we compared the thyroid autoantibody positivity rates among TS patients across different age groups (10–20 years, 20–30 years, and 30–40 years). The results demonstrated no statistically significant differences in thyroid autoantibody positivity rates between these age groups (**Table 4**). Furthermore, among TS patients with positive thyroid autoantibodies, there were no statistically significant differences in the rate of thyroid dysfunction across the different age groups (**Table 5**).

**Table 4:** The prevalence of AITD according to age.

	Number of negative thyroid autoantibodies [n (%)]	Number of positive thyroid autoantibodies [n (%)]	χ2	*P*
Age			1.15	0.56
10–20 years	12 (35.29%)	22 (64.71%)		
20–30 years	10 (38.46%)	16 (61.54%)		
30–40 years	2 (66.67%)	1 (33.33%)		

Prevalence of AITD is calculated over the total number of patients per age group.

**Table 5:** The prevalence of dysthyreosis according to age.

	Euthyroidism number [n (%)]	Dysthyreosis number [n (%)]	*P*
Age			0.20†
10–20 years	14 (63.64%)	8 (36.36%)	
20–30 years	14 (87.50%)	2 (12.50%)	
30–40 years	1 (100%)	0 (0%)	

Prevalence of dysthyreosis is calculated over the total number of patients per age group.

†Fisher’s exact test.

There were no significant differences in age, height, weight, BMI, TG, TC, HDL-C, LDL-C, Ca, P and FIns levels between the groups of negative and positive thyroid autoantibodies (**Table 6**). There were also no significant differences in age, height, weight, BMI, TG, TC, HDL-C, LDL-C, Ca, P and FIns levels between the groups of euthyroidism and dysthyreosis (**Table 7**).

**Table 6:** Factors associated with AITD.

Subject	Negative thyroid autoantibodies (n=24)	Negative thyroid autoantibodies (n=39)	t	*P*
Age (years)	21.07 ± 4.18	18.40 ± 3.026	1.851	0.07
Height (cm)	145.73 ± 8.26	146.77 ± 7.36	0.523	0.6
Weight (kg)	48.59 ± 12.25	47.27 ± 9.48	0.480	0.6
BMI (kg/m^2^)	22.66 ± 4.65	21.86 ± 3.76	0.750	0.45
TG (mmol/L)	1.52 ± 0.91	1.33 ± 0.58	1.744	0.09
TC (mmol/L)	5.34 ± 1.04	4.83 ± 1.17	0.559	0.6
HDL-C (mmol/L)	1.57 ± 0.32	1.53 ± 0.29	1.423	0.2
LDL-C (mmol/L)	3.11 ± 0.93	2.75 ± 0.97	0.496	0.6
Ca (mmol/L)	2.19 ± 0.36	2.23 ± 0.28	0.320	0.8
P (mmol/L)	1.43 ± 0.32	1.4 ± 0.22	0.146	0.9
FIns (uU/mL)	9.67 ± 5.97	9.85 ± 3.91	1.744	0.09

BMI, body mass index; TG, triglyceride; TC, total cholesterol; HDL-C, high density lipoprotein cholesterol; LDL-C, low density lipoprotein cholesterol; Ca, calcium; P, phosphorous; Fins, fasting insulin.

**Table 7:** Factors associated with dysthyreosis.

Subject	Euthyroidism (n=29)	Dysthyreosis (n=10)	t	*P*
Age (years)	20.21 ± 4.73	20.44 ± 4.10	-0.202	0.08
Height (cm)	146.79 ± 7.35	146.70 ± 7.79	0.035	0.9
Weight (kg)	46.97 ± 10.18	48.12 ± 7.47	-0.327	0.8
BMI (kg/m^2^)	21.68 ± 3.94	22.38 ± 3.30	-0.503	0.6
TG (mmol/L)	1.28 ± 0.52	1.46 ± 0.74	-0.851	0.4
TC (mmol/L)	4.87 ± 1.20	4.70 ± 1.14	0.393	0.7
HDL-C (mmol/L)	1.57 ± 0.31	1.40 ± 0.16	1.631	0.1
LDL-C (mmol/L)	2.76 ± 0.97	2.74 ± 1.02	0.048	0.9
Ca (mmol/L)	2.24 ± 0.26	2.20 ± 0.34	0.338	0.7
P (mmol/L)	1.37 ± 0.16	1.51 ± 0.33	-1.826	0.08
FIns (uU/mL)	9.45 ± 3.58	11.03 ± 4.78	-1.105	0.3

BMI, body mass index; TG, triglyceride; TC, total cholesterol; HDL-C, high density lipoprotein cholesterol; LDL-C, low density lipoprotein cholesterol; Ca, calcium; P, phosphorous; Fins, fasting insulin.

## 4 Discussion

Due to complete or partial deletion of the X chromosome, patients with TS are often affected by a variety of complications throughout their life, with autoimmune disorders (particularly thyroid dysfunction), congenital cardiovascular defects, atherosclerosis, osteoporosis and fractures, endocrine and metabolic disorders, hearing loss and specific cognitive deficits being recognized as contributing factors to the increased mortality and reduced life expectancy associated with TS (5, 23). Patients with TS have a higher risk of autoimmune thyroiditis than the normal population, not only during puberty and adulthood, but also in childhood, and 5%-10% of cases occur before puberty (24). Approximately 40% of TS patients are positive for thyroid autoantibodies, however, these patients rarely present with clinical symptoms of thyroid disease (8, 12, 25, 26). The majority of patients are diagnosed with subclinical hypothyroidism, with about 1/3 of them developing Hashimoto’s thyroiditis (26).

In the TS cohort of this study, the rate of AITD was 61.90%, which was higher than the rate of TS women in Europe and the United States, which was 39.4% and 43.7%, respectively (7). However, it was comparable to the rate of TS women in Japan (57%) (27). Differences in rates among different studies may be due to the differences in age, karyotype, sample size, and race of the included subjects. In this study, of the 39 TS women who were positive for thyroid autoantibodies, 7 developed hypothyroidism and 3 developed hyperthyroidism, whereas none of the 24 TS women who were negative for thyroid autoantibodies had thyroid dysfunction, suggesting that TS women who were positive for thyroid autoantibodies have a significantly increased risk of developing thyroid dysfunction, as reported in previous studies (27, 28). However, those women with TS who were negative for thyroid autoantibodies are still at risk for subclinical hypothyroidism (26). Therefore, negative thyroid autoantibodies do not completely exclude the possibility of future thyroid dysfunction. As a result, it is important to regularly check thyroid function in TS women, even if thyroid autoantibodies are negative, and appropriate treatment should be initiated promptly to prevent severe thyroid dysfunction.

It had been suggested that the increase in the long arm (Xq) and the loss of the short arm (Xp) of the X chromosome may play an important role in the development of autoimmunity (29, 30). Association of isochromosome X with excessive autoimmunity in TS patients suggested that genes on the long arm of the X chromosome (Xq) may play an important pathogenic role in the development of autoimmune diseases, and the imbalance between loci on the short and long arms of the X chromosome may increase the risk of immune dysfunction (16). FOXP3 which maps to the Xp11.23 locus of the short arm is critical for regulatory T cells function (31). The higher incidence of autoimmune diseases in individuals with X chromosome disorders, as well as in females, suggests that disruptions in the process of X chromosome inactivation may lead to reduced exposure to autoantigens (32). Furthermore, other immune-mediated diseases such as inflammatory bowel disease also occur more frequently in TS patients with isochromosome X (14, 15, 33). In contrast, a study in Taiwan showed that TS patients with a ring X chromosome had a higher risk of developing AITD (34). However, some studies suggested that the risk of AITD in TS patients was not related to karyotype (24, 26, 35, 36). The current study shows that AITD can occur in any type of karyotype, but patients with isochromosome X have an increased risk of AITD, as reported in previous studies (8, 12, 16, 37–44). There was no significant difference in the rate of thyroid dysfunction between karyotypes, which further supports the results of a previous study (45). One report emphasized that the incidence of AITD is related not only to genetic factors associated with the sex chromosome but also to hormone and environmental factors (46).

The different results of the above studies suggest that karyotype is not the only factor determining the rate of AITD in women with TS. The absence of the second X chromosome associated with ovarian dysfunction may further the autoimmune process in TS patients with the superimposed effects of environment, age, life habits and metabolic syndrome.

In the general population, with time euthyroid patients progress to hypothyroidism and thus the prevalence of hypothyroidism is higher in elderly patients (47). A previous study reported that women with the lowest ovarian reserves had higher levels of TPOAb, and the levels of the antibody also tended to increase over time, suggesting that the group had an increased risk of hypothyroidism with age (48). Regarding the correlation between TS combined with AITD and age, it was found that patients in the positive thyroid autoantibodies were significantly older compared to TS patients in the negative thyroid autoantibodies group (25, 27, 36). The risk of autoimmune diseases increases progressively with age, possibly due to a decrease in immune tolerance, often involving a decline in the integrity of the immune system for both innate and adaptive immune responses, a process known as immune senescence (49). Although several pathways involved in immune tolerance have not been fully elucidated, age-related declines in the number and function of specific B-cell subtypes, with a concomitant decrease in regulatory roles in the immune response, may lead to reduced immune tolerance and increased autoimmunity (50). Therefore, the likelihood of abnormal thyroid function is high in TS patients with age. In our TS patients, there was no difference in age between the negative thyroid autoantibodies and positive thyroid autoantibodies groups. In the positive thyroid autoantibodies group, there was also no difference in age between the euthyroidism group and the dysthyreosis group. The difference may be attributed to the fact that our study encompassed a narrower age range of 14–34 years for women with TS. In contrast, the studies conducted in Denmark and Japan included a broader age range of TS patients, spanning from 6–60 and 15–61 years, respectively (25, 27). Therefore, we hypothesize that the reasons for the different results between our study and previous studies are due to differences in genetics, environmental factors and lifestyle and dietary habits with Asian and European populations, in addition to the different age structure of the study population.

There is a potential association between multiple metabolic abnormalities and AITD. Studies have shown that AITD can be caused by obesity, malnutrition, nutrient deficiencies and abnormal glucose metabolism (17). The etiology of the changes in thyroid function that can be influenced by obesity is still unclear. However, several mechanisms have been proposed including the adaptive process to increase energy expenditure, hyperleptinemia, changes in the activity of deiodinases, thyroid hormone resistance, chronic low-grade inflammation, and insulin resistance (51). A study of obesity and thyroid disease found that the rate of goiter and thyroid nodules increased with increasing BMI and was highest in the obese group with unhealthy metabolism (52). Insulin resistance and hyperinsulinaemia associated with obesity have a mild but persistent stimulatory effect on the proliferation of thyroid cells, leading to an increase in the size of the thyroid gland and the formation of hyperplastic nodules (53). In addition, impaired metabolism of minerals like calcium, phosphorus, magnesium and copper can lead to AITD. A previous study reported that mean serum calcium, magnesium and copper levels were significantly decreased while serum phosphorus levels were increased in hypothyroid cases as compared to euthyroid cases (54). However, our literature search revealed that while numerous scholars have studied obesity, abnormal glucose metabolism, and other thyroid-related disorders in the population, few scholars have focused on studying similar traits in TS patients. In our study, we found that there was no significant correlation between the occurrence of AITD and BMI, calcium and phosphorus levels, or FIns level in TS patients. Larger cohort studies with an expanded age range are warranted to further examine these parameters and their association with AITD in TS patients.

Previous studies have mostly shown that thyroid hormones play an important role in lipid metabolism and that hypothyroidism can lead to dyslipidemia (55–57). A longitudinal cohort study in Korea showed that dyslipidemia is a risk factor for hypothyroidism in women (18). Notably, dyslipidemia can contribute to the development and progression of other autoimmune diseases, such as SLE and IgA nephropathy (58, 59). Furthermore, several studies have reported that lipid-reducing agents relieve the severity of autoimmunity (60, 61). A previous study has indicated that a woman with TS and combined hypothyroidism exhibited TC levels twice as high as normal and TG levels four times as high as normal. Following thyroid hormone replacement therapy, her lipid levels returned to normal values (24). The results of this study found that comorbid AITD in women with TS was not associated with TG, TC, HDL-C and LDL-C levels. This is similar to the result of the Danish study of adults with TS that failed to find any correlation between dyslipidemia and hypothyroidism in TS patients (62).

The present study is one of the few studies on whether BMI, calcium and phosphorus levels, lipids and fasting insulin are associated with AITD in women with TS. Due to limitations of the original data, our study failed to explore the association of vitamin D levels, socioeconomic status, education, occupation, dietary preference, outdoor physical activity, growth hormone therapy and estrogen replacement therapy with AITD in TS patients. A further limitation is the relatively low sample size of TS patients with AITD, which may have contributed to the negative findings in terms of risk factors and AITD in TS. Prospective multicenter multidimensional studies with relatively large samples are needed in the future to study AITD and thyroid dysfunction in TS patients. Correlations between age and karyotype and AITD in TS patients are now well established. Therefore, we need to turn our focus onto the pathogenesis of AITD in TS patients. This will allow for exploration of effective clinical treatment options and development more comprehensive and effective management strategies.

## 5 Conclusions

The rate of AITD in our TS patients was 61.90% (39/63), of which the rate of thyroid dysfunction was 15.87% (10/63). Compared with other karyotypes, patients with isochromosome X have an increased risk of AITD. In addition, patients with positive thyroid autoantibodies have a higher risk of thyroid dysfunction. For the diagnosed children with TS, endocrinologists should pay attention to their thyroid function. It is suggested that routine thyroid examinations be carried out regularly every year in order to detect thyroid abnormalities early and start appropriate treatment in time, so as to avoid possible effects on growth, intellectual development and even the cardiovascular system.

## Data Availability

The original contributions presented in the study are included in the article/supplementary material. Further inquiries can be directed to the corresponding author.

## References

[1] Cameron-Pimblett A La Rosa C King TFJ Davies MC Conway GS. The Turner syndrome life course project: Karyotype-phenotype analyses across the lifespan. Clin Endocrinol (Oxf). (2017) 87:532–8. doi: 10.1111/cen.13394, PMID: 28617979

[2] Gravholt CH Andersen NH Conway GS Dekkers OM Geffner ME Klein KO. Clinical practice guidelines for the care of girls and women with Turner syndrome: proceedings from the 2016 Cincinnati International Turner Syndrome Meeting. Eur J Endocrinol. (2017) 177:G1–g70. doi: 10.1530/EJE-17-0430, PMID: 28705803

[3] ALvarez-Nava F Soto M Sánchez MA Fernández E Lanes R. Molecular analysis in Turner syndrome. J Pediatr. (2003) 142:336–40. doi: 10.1067/mpd.2003.95, PMID: 12640385

[4] Rivkees S. Beyond the karyotype: are new screening methods needed for girls with Turner’s syndrome? J Pediatr Endocrinol Metab. (2006) 19:1093–4. doi: 10.1515/jpem.2006.19.9.1093, PMID: 17128556

[5] Bianchi I Lleo A Gershwin ME Invernizzi P. The X chromosome and immune associated genes. J Autoimmun. (2012) 38:J187–92. doi: 10.1016/j.jaut.2011.11.012, PMID: 22178198

[6] Atria A Sanz R Donoso S. Necropsy study of a case of Turner’s syndrome; a case report. J Clin Endocrinol Metab. (1948) 8:397–405. doi: 10.1210/jcem-8-5-397, PMID: 18863971

[7] Mohamed SOO Elkhidir IHE Abuzied AIH Noureddin AAMH Ibrahim GAA Mahmoud AAA. Prevalence of autoimmune thyroid diseases among the Turner Syndrome patients: meta-analysis of cross sectional studies. BMC Res Notes. (2018) 11:842. doi: 10.1186/s13104-018-3950-0, PMID: 30486859 PMC6264051

[8] Bakalov VK Gutin L Cheng CM Zhou J Sheth P Shah K. Autoimmune disorders in women with turner syndrome and women with karyotypically normal primary ovarian insufficiency. J Autoimmun. (2012) 38:315–21. doi: 10.1016/j.jaut.2012.01.015, PMID: 22342295 PMC3358475

[9] Wegiel M Antosz A Gieburowska J Szeliga K Hankus M Grzybowska-Chlebowczyk U. Autoimmunity predisposition in girls with turner syndrome. Front Endocrinol (Lausanne). (2019) 10:511. doi: 10.3389/fendo.2019.00511, PMID: 31417494 PMC6682631

[10] Pessach IM Notarangelo LD. X-linked primary immunodeficiencies as a bridge to better understanding X-chromosome related autoimmunity. J Autoimmun. (2009) 33:17–24. doi: 10.1016/j.jaut.2009.03.003, PMID: 19361956

[11] Aversa T Lombardo F Valenzise M Messina MF Sferlazzas C Salzano G. Peculiarities of autoimmune thyroid diseases in children with Turner or Down syndrome: an overview. Ital J Pediatr. (2015) 41:39. doi: 10.1186/s13052-015-0146-2, PMID: 25971674 PMC4440559

[12] Grossi A Crinò A Luciano R Lombardo A Cappa M Fierabracci A. Endocrine autoimmunity in Turner syndrome. Ital J Pediatr. (2013) 39:79. doi: 10.1186/1824-7288-39-79, PMID: 24355069 PMC3901035

[13] Jørgensen KT Rostgaard K Bache I Biggar RJ Nielsen NM Tommerup N. Autoimmune diseases in women with Turner’s syndrome. Arthritis Rheum. (2010) 62:658–66. doi: 10.1002/art.27270, PMID: 20187158

[14] Hayward PA Satsangi J Jewell DP. Inflammatory bowel disease and the X chromosome. Qjm. (1996) 89:713–8. doi: 10.1093/qjmed/89.9.713, PMID: 8917748

[15] Price WH. A high incidence of chronic inflammatory bowel disease in patients with Turner’s syndrome. J Med Genet. (1979) 16:263–6. doi: 10.1136/jmg.16.4.263, PMID: 490578 PMC1012667

[16] Elsheikh M Wass JA Conway GS. Autoimmune thyroid syndrome in women with Turner’s syndrome–the association with karyotype. Clin Endocrinol (Oxf). (2001) 55:223–6. doi: 10.1046/j.1365-2265.2001.01296.x, PMID: 11531929

[17] Kawicka A Regulska-Ilow B Regulska-Ilow B. Metabolic disorders and nutritional status in autoimmune thyroid diseases. Postepy Hig Med Dosw (Online). (2015) 69:80–90. doi: 10.5604/17322693.1136383, PMID: 25614676

[18] Kim HI Kim TH Kim H Kim SW Hahm JR Chung JH. Dyslipidemia is a risk factor for hypothyroidism in women: A longitudinal cohort study from South Korea. Thyroid. (2023) 33:100–8. doi: 10.1089/thy.2022.0216, PMID: 36602760

[19] Spilack AM Goulart AC Janovsky CCPS de Almeida-Pittito B Lotufo PA Sommer Bittencourt M. The impact of diabetes and subclinical hypothyroidism association with coronary artery calcium: results from the Brazilian Longitudinal Study of Adult Health (ELSA-Brasil). Arch Endocrin Metab. (2023) 68:e220375. doi: 10.20945/2359-4292-2022-0375, PMID: 37988662 PMC10916802

[20] Arambam P Gupta S Kaul U Ranjan P Sekhawat S Janardhanan R. Hypothyroidism in acute coronary syndrome - A prospective Indian study. Indian Heart J. (2024) 76:44–7. doi: 10.1016/j.ihj.2023.12.008, PMID: 38296053 PMC10943535

[21] Hjerrild BE Mortensen KH Gravholt CH. Turner syndrome and clinical treatment. Br Med Bull. (2008) 86:77–93. doi: 10.1093/bmb/ldn015, PMID: 18400842

[22] Wolff DJ Van Dyke DL Powell CM. Laboratory guideline for Turner syndrome. Genet Med. (2010) 12:52–5. doi: 10.1097/GIM.0b013e3181c684b2, PMID: 20081420

[23] Lin H Wang X Qin S Luo F Cen Y Lash GE. Incidence and risk factors of hearing loss in patients with Turner Syndrome. Front Public Health. (2023) 11:1076812. doi: 10.3389/fpubh.2023.1076812, PMID: 36998272 PMC10043252

[24] El-Mansoury M Bryman I Berntorp K Hanson C Wilhelmsen L Landin-Wilhelmsen K. Hypothyroidism is common in turner syndrome: results of a five-year follow-up. J Clin Endocrinol Metab. (2005) 90:2131–5. doi: 10.1210/jc.2004-1262, PMID: 15623818

[25] Mortensen KH Cleemann L Hjerrild BE Nexo E Locht H Jeppesen EM. Increased prevalence of autoimmunity in Turner syndrome–influence of age. Clin Exp Immunol. (2009) 156:205–10. doi: 10.1111/j.1365-2249.2009.03895.x, PMID: 19298606 PMC2759466

[26] Gawlik A Gawlik T Januszek-Trzciakowska A Patel H Malecka-Tendera E. Incidence and dynamics of thyroid dysfunction and thyroid autoimmunity in girls with Turner’s syndrome: a long-term follow-up study. Horm Res Paediatr. (2011) 76:314–20. doi: 10.1159/000331050, PMID: 21997702

[27] Fukuda I Hizuka N Kurimoto M Morita J Tanaka S Yamakado Y. Autoimmune thyroid diseases in 65 Japanese women with Turner syndrome. Endocr J. (2009) 56:983–6. doi: 10.1507/endocrj.k09e-141, PMID: 19671996

[28] Yu BS Chen RH Guo XR Zhang YQ Wang XN Li Z. Thyroid autoantibodies in children with Turner’s syndrome. Zhonghua Er Ke Za Zhi. (2005) 43:96–8., PMID: 15833160

[29] Wilson R Chu CE Donaldson MD Thomson JA McKillop JH Connor JM. An increased incidence of thyroid antibodies in patients with Turner’s syndrome and their first degree relatives. Autoimmunity. (1996) 25:47–52. doi: 10.3109/08916939608994725, PMID: 9161699

[30] Ivarsson SA Ericsson UB Nilsson KO Gustafsson J Hagenäs L Häger A. Thyroid autoantibodies, Turner’s syndrome and growth hormone therapy. Acta Paediatr. (1995) 84:63–5. doi: 10.1111/j.1651-2227.1995.tb13485.x, PMID: 7734902

[31] Zinn AR Tonk VS Chen Z Flejter WL Gardner HA Guerra R. Evidence for a Turner syndrome locus or loci at Xp11.2-p22.1. Am J Hum Genet. (1998) 63:1757–66. doi: 10.1086/302152, PMID: 9837829 PMC1377648

[32] Chitnis S Monteiro J Glass D Apatoff B Salmon J Concannon P. The role of X-chromosome inactivation in female predisposition to autoimmunity. Arthritis Res. (2000) 2:399–406. doi: 10.1186/ar118, PMID: 11056674 PMC17816

[33] Bakalov VK Cheng C Zhou J Bondy CA. X-chromosome gene dosage and the risk of diabetes in Turner syndrome. J Clin Endocrinol Metab. (2009) 94:3289–96. doi: 10.1210/jc.2009-0384, PMID: 19567529 PMC2741724

[34] Chang P Tsai WY Hou JW Hsiao PH Lee JS. Autoimmune thyroiditis in children with Turner syndrome. J Formos Med Assoc. (2000) 99:823–6. 11155770

[35] Witkowska-Sędek E Borowiec A Kucharska A Chacewicz K Rumińska M Demkow U. Thyroid autoimmunity in girls with turner syndrome. Adv Exp Med Biol. (2017) 1022:71–6. doi: 10.1007/5584_2017_42, PMID: 28456931

[36] Chen RM Zhang Y Yang XH Lin XQ Yuan X. Thyroid disease in Chinese girls with Turner syndrome. J Pediatr Endocrinol Metab. (2015) 28:201–5. doi: 10.1515/jpem-2014-0199, PMID: 25229413

[37] Hamilton CR Jr. Moldawer M Rosenberg HS. Hashimoto’s thyroiditis and Turner’s syndrome. Arch Intern Med. (1968) 122:69–72. doi: 10.1515/jpem-2014-0199, PMID: 5694912

[38] Germain EL Plotnick LP. Age-related anti-thyroid antibodies and thyroid abnormalities in Turner syndrome. Acta Paediatr Scand. (1986) 75:750–5. doi: 10.1111/j.1651-2227.1986.tb10285.x, PMID: 3494376

[39] de Kerdanet M Lucas J Lemee F Lecornu M. Turner’s syndrome with X-isochromosome and Hashimoto’s thyroiditis. Clin Endocrinol (Oxf). (1994) 41:673–6. doi: 10.1111/j.1365-2265.1994.tb01835.x, PMID: 7828358

[40] Gruñeiro de Papendieck L Iorcansky S Coco R Rivarola MA Bergadá C. High incidence of thyroid disturbances in 49 children with Turner syndrome. J Pediatr. (1987) 111:258–61. doi: 10.1016/s0022-3476(87)80082-3, PMID: 3612400

[41] Chiovato L Larizza D Bendinelli G Tonacchera M Marinó M Mammoli C. Autoimmune hypothyroidism and hyperthyroidism in patients with Turner’s syndrome. Eur J Endocrinol. (1996) 134:568–75. doi: 10.1530/eje.0.1340568, PMID: 8664977

[42] Hamza RT Raof NA Abdallah KO. Prevalence of multiple forms of autoimmunity in Egyptian patients with Turner syndrome: relation to karyotype. J Pediatr Endocrinol Metab. (2013) 26:545–50. doi: 10.1515/jpem-2012-0265, PMID: 23446949

[43] Yeşilkaya E Bereket A Darendeliler F Baş F Poyrazoğlu Ş Küçükemre Aydın B. Turner syndrome and associated problems in Turkish children: a multicenter study. J Clin Res Pediatr Endocrinol. (2015) 7:27–36. doi: 10.4274/jcrpe.1771, PMID: 25800473 PMC4439889

[44] Lee YL Zaini AA Idris AN Abdullah RA Wong JS Hong JS. Thyroid autoimmunity and autoimmune thyroid disease in Malaysian girls with Turner syndrome: An understudied population. J Paediatr Child Health. (2023) 59:879–84. doi: 10.1111/jpc.16405, PMID: 37066819

[45] Katahira M Tsunekawa T Mizoguchi A Yamaguchi M Tsuru K Takashima H. Clinical significance of thyroglobulin antibodies and thyroid peroxidase antibodies in Graves’ disease: a cross-sectional study. Hormones (Athens). (2023) 22:253–61. doi: 10.1007/s42000-023-00437-7, PMID: 36811756

[46] Kucharska A. Sex and autoimmune thyroid diseases. Pediatr Endocrinol. (2014) 13:57–64. doi: 10.18544/ep-01.13.02.1486

[47] Amino N. Autoimmunity and hypothyroidism. Baillieres Clin Endocrinol Metab. (1988) 2:591–617. doi: 10.1016/s0950-351x(88)80055-7, PMID: 3066320

[48] Bahri S Tehrani FR Amouzgar A Rahmati M Tohidi M Vasheghani M. Overtime trend of thyroid hormones and thyroid autoimmunity and ovarian reserve: a longitudinal population study with a 12-year follow up. BMC Endocr Disord. (2019) 19:47. doi: 10.1186/s12902-019-0370-7, PMID: 31064360 PMC6505305

[49] Lindstrom TM Robinson WH. Rheumatoid arthritis: a role for immunosenescence? J Am Geriatr Soc. (2010) 58:1565–75. doi: 10.1111/j.1532-5415.2010.02965.x, PMID: 20942872 PMC3055796

[50] Duggal NA Upton J Phillips AC Sapey E Lord JM. An age-related numerical and functional deficit in CD19(+) CD24(hi) CD38(hi) B cells is associated with an increase in systemic autoimmunity. Aging Cell. (2013) 12:873–81. doi: 10.1111/acel.12114, PMID: 23755918 PMC3814412

[51] Fontenelle LC Feitosa MM Severo JS Freitas TE Morais JB Torres-Leal FL. Thyroid function in human obesity: underlying mechanisms. Horm Metab Res. (2016) 48:787–94. doi: 10.1055/s-0042-121421, PMID: 27923249

[52] Song B Lu C Teng D Shan Z Teng W. Association between different metabolic phenotypes of obesity and thyroid disorders among Chinese adults: a nationwide cross-sectional study. Front Endocrinol (Lausanne). (2023) 14:1158013. doi: 10.3389/fendo.2023.1158013, PMID: 37152970 PMC10162017

[53] Tsatsoulis A. The role of insulin resistance/hyperinsulinism on the rising trend of thyroid and adrenal nodular disease in the current environment. J Clin Med. (2018) 7:37. doi: 10.3390/jcm7030037, PMID: 29495350 PMC5867563

[54] Athokpham D Mohanty S Pinnelli V. Status of serum calcium, phosphorus, magnesium and copper in hypothyroid patients- A case control study. J Clin Diagn Res. (2020). 14:BC05–8. doi: 10.7860/jcdr/2020/44711.14094

[55] Siscart J Perejón D Serna MC Oros M Godoy P Sole E. Prevalence, risk factors, and consequences of hypothyroidism among pregnant women in the health region of Lleida: A cohort study. PloS One. (2023) 18:e0278426. doi: 10.1371/journal.pone.0278426, PMID: 37831694 PMC10575516

[56] Su X Chen X Wang B. Relationship between the development of hyperlipidemia in hypothyroidism patients. Mol Biol Rep. (2022) 49:11025–35. doi: 10.1371/journal.pone.0278426, PMID: 36097119

[57] S S Mb V. A Study of Lipid Profile in Patients with Subclinical Hypothyroidism. J Assoc Physicians India. (2022) 70(4):11–2.

[58] Zhuang H Lin Z Zeng S Jiang M Chen L Jiang X. Dyslipidemia may be a risk factor for progression in children with IgA nephropathy. Pediatr Nephrol. (2022) 37:3147–56. doi: 10.1007/s00467-022-05480-x, PMID: 35347403

[59] Huang S Zhang Z Cui Y Yao G Ma X Zhang H. Dyslipidemia is associated with inflammation and organ involvement in systemic lupus erythematosus. Clin Rheumatol. (2023) 42:1565–72. doi: 10.1007/s10067-023-06539-2, PMID: 36790644 PMC10203001

[60] Wierzbicki AS Viljoen A. Hyperlipidaemia in paediatric patients: the role of lipid-lowering therapy in clinical practice. Drug Saf. (2010) 33:115–25. doi: 10.2165/11319490-000000000-00000, PMID: 20082538

[61] Yu HH Chen PC Yang YH Wang LC Lee JH Lin YT. Statin reduces mortality and morbidity in systemic lupus erythematosus patients with hyperlipidemia: A nationwide population-based cohort study. Atherosclerosis. (2015) 243:11–8. doi: 10.1016/j.atherosclerosis.2015.08.030, PMID: 26342937

[62] Gravholt CH Christian Klausen I Weeke J Sandahl Christiansen J. Lp(a) and lipids in adult Turner’s syndrome: impact of treatment with 17beta-estradiol and norethisterone. Atherosclerosis. (2000) 150:201–8. doi: 10.1016/s0021-9150(99)00369-x, PMID: 10781652

